# Decoration of Pt on Cu/Co double-doped CeO_2_ nanospheres and their greatly enhanced catalytic activity†
†Electronic supplementary information (ESI) available. See DOI: 10.1039/c5sc04069h


**DOI:** 10.1039/c5sc04069h

**Published:** 2015-11-25

**Authors:** Fan Wang, Wang Li, Xilan Feng, Dapeng Liu, Yu Zhang

**Affiliations:** a Key Laboratory of Bio-Inspired Smart Interfacial Science and Technology of Ministry of Education , School of Chemistry and Environment , Beihang University , Beijing 100191 , P.R. China . Email: liudp@buaa.edu.cn ; Email: jade@buaa.edu.cn; b State Key Laboratory of Rare Earth Resource Utilization , Changchun Institute of Applied Chemistry , Chinese Academy of Science , Changchun , 130022 Jilin , China

## Abstract

In this paper, we report an efficient strategy for the synthesis of Cu/Co double-doped CeO_2_ nanospheres (Cu_*x*_Co_1–*x*_–CeO_2_–Pt, 0 ≤ *x* ≤ 1), which were fabricated *via* a simple water–glycol system. The following *in situ* surface decoration of Pt nanoparticles make these nanospheres highly active for the catalytic reduction of nitrophenol and CO oxidation. Detailed tests show that their catalytic performance strongly depends on the doping components and ion concentration of Cu and Co ions. The best samples of Cu_0.50_Co_0.50_–CeO_2_–Pt and Cu_0.34_Co_0.66_–CeO_2_–Pt demonstrate an excellent turnover frequency (TOF) of more than 450 h^–1^ after five cycles and retains about 99% conversion by using NH_3_BH_3_ as a reductant to reduce nitrophenol. Moreover, Cu_0.50_Co_0.50_–CeO_2_–Pt possesses a much lower light-off and *T*_100_ (the temperature for 100% CO oxidation) temperature compared with the other catalysts.

## Abstract 2

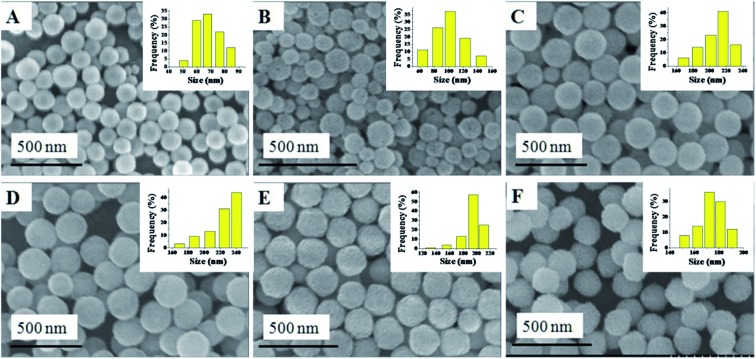
In this paper, we report an efficient strategy for the synthesis of Cu/Co double-doped CeO_2_ nanospheres (Cu_*x*_Co_1–*x*_–CeO_2_–Pt, 0 ≤ *x* ≤ 1), which were fabricated *via* a simple water–glycol system.

## Introductions

Among the metal oxides, CeO_2_ has attracted great attention due to its excellent physicochemical properties including its good optical properties, mechanical strength, oxygen ion conductivity, and high thermal stability. CeO_2_ has a fluorite-like cubic structure with each Ce^4+^ ion surrounded by eight O^2–^ ions in a face-centered cubic (fcc) arrangement, whereas each O^2–^ ion is tetrahedrally surrounded by four Ce^4+^ ions. Intrinsic oxygen vacancy defects can be rapidly formed and eliminated in the lattice of CeO_2_, which favors mediation of lattice expansion and strain, and hence contributes significantly towards stable grain boundary structures. Hence, CeO_2_ has been successfully employed in various applications such as in energy and magnetic data storage,^1^ photocatalytic applications,^2^–^4^ as sensors for CO,^5^–^9^ H_2_O_2_,^10^ NH_3_,^11^ and nitrophenol,^12^,^13^ as a UV blocker,^14^ in solar fuel synthesis,^15^ water oxidation,^16^,^17^ oxygen transfer,^18^ fuel cells,^19^,^20^ gates for metal-oxide semiconductor devices,^21^ as a promoter in three-way catalysts for the elimination of polluted auto-exhaust gases from vehicles,^22^–^24^ and so on.

The larger the amount of oxygen vacancies that CeO_2_ possesses, the more efficient it will be for storing oxygen. Thus, it seems meaningful to control the generation of oxygen vacancies to improve its physicochemical properties. Endeavours have been devoted to introduce dopants to improve the catalytic performance of CeO_2_. It has been reported that its catalytic activity can be considerably enhanced by tuning the surface and interface structure through doping with isovalent/aliovalent cations into the CeO_2_ lattice.^25^–^30^ The isovalent cations that are frequently used are Ti^4+^, Zr^4+^, Hf^4+^, and Sn^4+^, while aliovalent cations used include Mn^2+^, Ni^2+^, Zn^2+^, Ca^2+^, Mn^3+^, Sc^3+^, Y^3+^, Gd^3+^, Sm^3+^, Eu^3+^, La^3+^, *etc.* The substitution of isovalent dopants into the CeO_2_ lattice decreases the oxygen vacancy formation energy due to structural distortion, whereas in the case of aliovalent dopants, the decrease in the defect formation energy is due to structural distortion as well as electronic modification, resulting in the generation of extra oxygen vacancies.^31^,^32^ So once noble metals or metal oxides form hybrids with CeO_2_, they often exhibit greatly enhanced catalytic activity, stability and selectivity.

Noble metal nanoparticles have been extensively studied for decades due to their high performance in many kinds of catalytic reactions. Smaller sized noble metal nanoparticles often have a larger fraction of exposed atoms on the particle surface, demonstrating better catalytic activity. Moreover, the strong synergistic effect between noble metals and the support greatly favors improvement of the catalytic performance,^33^–^38^ especially for Pt–CeO_2_ systems.^39^–^44^ A recent report by Chowdhury *et al.*, reveals that doping can influence the surface acid–base properties of mesoporous CeO_2_ and its catalytic behavior.^43^ It is highly expected that incorporation of the cations into a ceria lattice structure will influence the redox properties of ceria in favour of synergistic interactions with noble metal nanoparticles towards enhanced activity for catalytic nitrophenol reduction and CO oxidation.

In this paper, an efficient strategy was developed for the synthesis of Cu_*x*_Co_1–*x*_–CeO_2_–Pt hybrids. First, double-doped CeO_2_ nanospheres were fabricated in a water–glycol mixed system, followed by a self-assembly process to *in situ* deposit Pt nanoparticles on the surface of the as-obtained double-doped CeO_2_ nanospheres to form the final hybrids. Then, the as-obtained catalysts with different doping components were studied in detail to find the optimal doping ratios with the best catalytic performance for the reduction of 4-NP (4-nitrophenol) by NH_3_BH_3_ and the oxidation of CO. Furthermore, a detailed discussion has been made to clarify the role of the doping elements in the reduction of 4-NP and oxidation of CO, according to the analytical results.

## Experimental section

### Synthesis of CeO_2_ nanospheres:

The synthesis of CeO_2_ nanospheres was carried out by a previously reported method.^45^ 500 mg of Ce(NO_3_)_3_·6H_2_O and 200 mg of PVP were dissolved in 14 mL of ethylene glycol, and then 1 mL of deionized water was added to the above solution. After continuous stirring for 30 min, the clear solution was transferred into a Teflon-lined autoclave of 20 mL capacity and heated for 8 h at 160 °C. When the autoclave was cooled at room temperature, the products were collected and washed with deionized water and absolute alcohol several times. Finally, the products were dried at 60 °C overnight, and then calcined at 300 °C for 1 h at 1 °C min^–1^.

### Synthesis of Cu_*x*_Co_1–*x*_–CeO_2_ nanospheres

500 mg of Ce(NO_3_)_3_·6H_2_O and 200 mg of PVP were dissolved in 14 mL of ethylene glycol, and then 0.66 mL of 20 mg mL^–1^ CuCl_2_·2H_2_O and 0.34 mL of a 20 mg mL^–1^ CoCl_2_·2H_2_O solution were added to the above solution. The mixed solutions were transferred into a Teflon-lined autoclave of 20 mL capacity and heated for 8 h at 160 °C. When the autoclave was cooled to room temperature, the products were collected and washed with deionized water and absolute alcohol several times. Finally, the products were dried at 60 °C overnight, and then calcined at 300 °C at 1 °C min^–1^ for 1 h. The above products were labeled as Cu_0.66_Co_0.34_–CeO_2_. Cu–CeO_2_, Cu_0.50_Co_0.50_–CeO_2_, Cu_0.34_Co_0.66_–CeO_2_ and Co–CeO_2_ were prepared in a similar process, except for changing the CuCl_2_·2H_2_O/CoCl_2_·2H_2_O (v/v) to 1 mL CuCl_2_·2H_2_O and 0 mL CoCl_2_·2H_2_O, 0.5 mL CuCl_2_·2H_2_O and 0.5 mL CoCl_2_·2H_2_O, 0.34 mL CuCl_2_·2H_2_O and 0.66 mL CoCl_2_·2H_2_O, and 0 mL CuCl_2_·2H_2_O and 1 mL CoCl_2_·2H_2_O, respectively.

### Synthesis of Cu_*x*_Co_1–*x*_–CeO_2_–Pt hybrids

70 mg of Cu_*x*_Co_1–*x*_–CeO_2_ nanospheres and 42 mg of PVP were first dissolved in 60 mL of ethylene glycol. After that, 0.84 mL of 0.02 M K_2_PtCl_4_ aqueous solution was added to the above solution. Then, the mixture was heated to 110 °C and was maintained at this temperature for 2 h. The product was collected by centrifugation and washed with deionized water several times and dried in an oven.

### Characterization

The X-ray diffraction patterns of the products were collected on a Rigaku-D/max 2500 V X-ray diffractometer with Cu_Kα_ radiation (*λ* = 1.5418 Å), with an operation voltage and current maintained at 40 kV and 40 mA. Transmission electron microscopic (TEM) images were obtained with a TECNAI G2 high-resolution transmission electron microscope operating at 200 kV. Inductively coupled plasma (ICP) analyses were performed with a Varian Liberty 200 spectrophotometer to determine the contents. X-ray photoelectron spectroscopy (XPS) measurements were taken on an ESCALAB-MKII 250 photoelectron spectrometer (VG Co.) with Al_Kα_ X-ray radiation as the X-ray source for excitation. Decreases in the concentration of 4-NP were analyzed by UV-vis-NIR (SHIMADZU, UV-3600) spectrophotometer. The catalytic performances of the catalysts for CO oxidation were monitored on-line by gas chromatography (GC9800).

### Catalytic tests

Chemical reduction of nitrophenol by NH_3_BH_3_: aqueous solutions of 4-NP (0.01 M) and NH_3_BH_3_ (0.1 M) were freshly prepared. 20 μL of the 4-NP solution and 100 μL of the NH_3_BH_3_ solution were added to a quartz cuvette containing 2 mL of water. Then, 20 μL of 5 mg mL^–1^ catalysts were injected into the cuvette to start the reaction. Since the spectrophotometer has a function to display the instant absorbance of a fixed absorption peak such as 400 nm, we can easily monitor the intensity of the absorption peak at 400 nm as a function of time. After each round of reaction, another 20 μL of 4-NP solution and 100 μL NH_3_BH_3_ aqueous solution were added to the reaction solution. This step was repeated 10 times to study the stability of the catalysts. The reduction of 4-NP by NH_3_BH_3_ can be briefly expressed as follows:
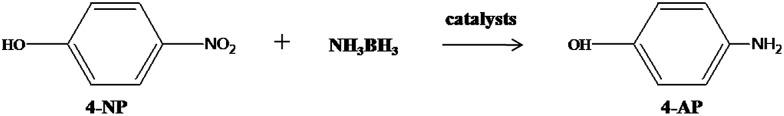


### CO oxidation

20 mg of catalysts were put in a stainless steel reaction tube. The CO oxidation tests were performed under conditions in 1% CO and 20% O_2_ in N_2_ at a total flow rate of 30 mL min^–1^, and a space velocity (SV) of 90 000 mL h^–1^ g_cat_^–1^. The composition of the gas was monitored on-line by gas chromatography.

## Results and discussion

**Fig. 1:**
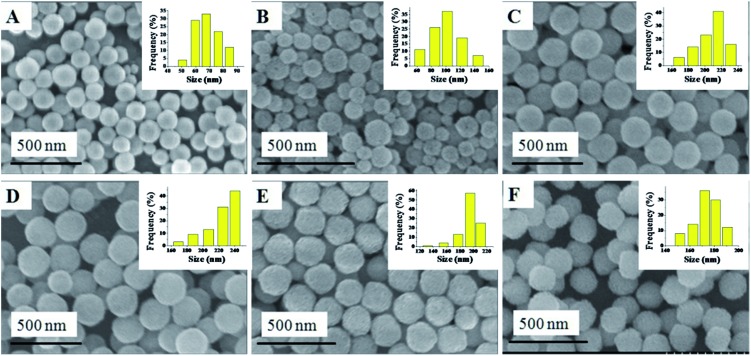
SEM images of (A) Cu–CeO_2_; (B) Cu_0.66_Co_0.34_–CeO_2_; (C) Cu_0.50_Co_0.50_–CeO_2_; (D) Cu_0.34_Co_0.66_–CeO_2_; (E) Co–CeO_2_; (F) CeO_2_.

**Fig. 2:**
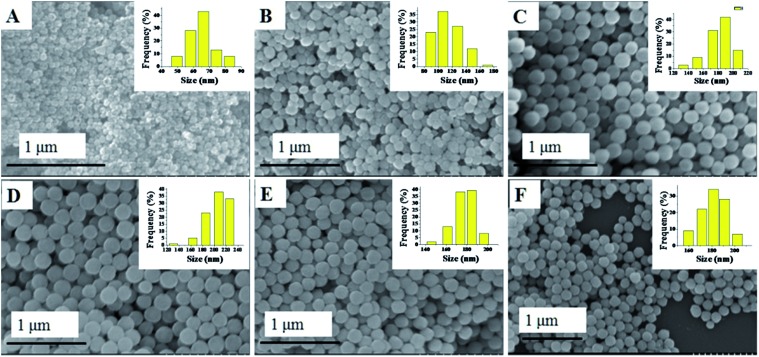
SEM images of (A) Cu–CeO_2_–Pt; (B) Cu_0.66_Co_0.34_–CeO_2_–Pt; (C) Cu_0.50_Co_0.50_–CeO_2_–Pt; (D) Cu_0.34_Co_0.66_–CeO_2_–Pt; (E) Co–CeO_2_–Pt; (F) CeO_2_–Pt.

The synthesis of the Cu_*x*_Co_1–*x*_–CeO_2_–Pt hybrid nanocatalysts involved two steps. Uniform Cu_*x*_Co_1–*x*_–CeO_2_ nanospheres were first acquired by tuning the doping concentration of Co^2+^ and Cu^2+^, and then they served as a support for the *in situ* deposition of Pt nanoparticles on their surface. Fig. 1 shows typical SEM images of the as-obtained Cu_*x*_Co_1–*x*_–CeO_2_ samples, in which some detailed morphological and structural features can be found. All these samples are sphere-like with no obvious fragments. Their size distributions are shown in Fig. 1 (inset) and are listed in Table S1.† Compared with the pure CeO_2_ nanospheres of 174 nm, the Cu-rich ones are much smaller and are even less than 100 nm, while those Co-rich samples show a bigger size around 200 nm. Fig. 2 shows the SEM images of the as-obtained samples after the addition of K_2_PtCl_4_ aqueous solution and after being refluxed at 110 °C for 2 h. However, it is hard for us to distinguish the differences of the surface from the Cu_*x*_Co_1–*x*_–CeO_2_ nanospheres, and no Pt particles can be found, indicating that the size of the deposited Pt nanoparticles should be very small in the Cu_*x*_Co_1–*x*_–CeO_2_–Pt hybrids. The sizes of the as-obtained Cu_*x*_Co_1–*x*_–CeO_2_–Pt hybrids are shown in Table S2.†

**Fig. 3:**
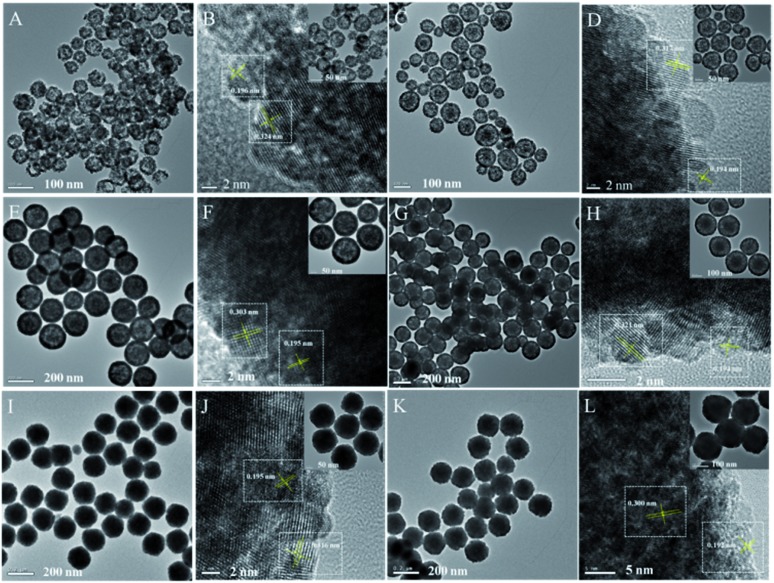
TEM images of (A) and (B) Cu–CeO_2_–Pt; (C) and (D) Cu_0.66_Co_0.34_–CeO_2_–Pt; (E) and (F) Cu_0.50_Co_0.50_–CeO_2_–Pt; (G) and (H) Cu_0.34_Co_0.66_–CeO_2_–Pt; (I) and (J) Co–CeO_2_–Pt; (K) and (L) CeO_2_–Pt.

**Fig. 4:**
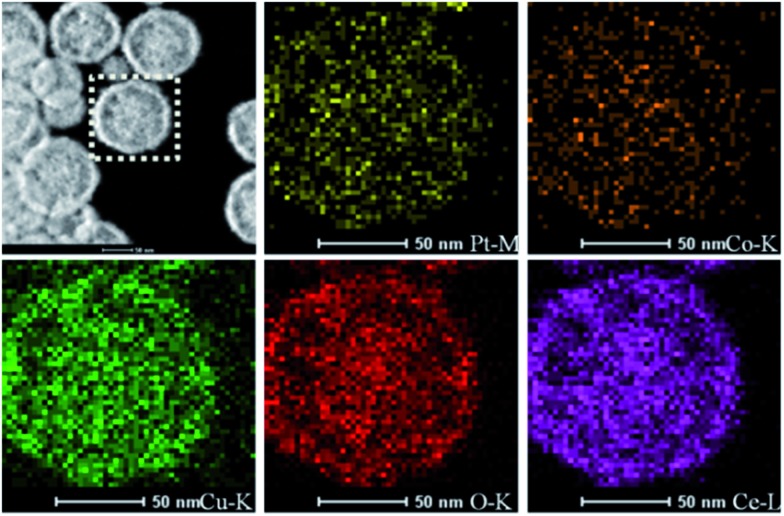
HAADF-STEM images of Cu_0.66_Co_0.34_–CeO_2_–Pt nanospheres.

TEM characterization can tell us more information about the final Cu_*x*_Co_1–*x*_–CeO_2_–Pt hybrids. As shown in Fig. 3, these Cu_*x*_Co_1–*x*_–CeO_2_–Pt hybrids maintained their initial morphologies and inner nanostructures well. With a decrease in the amount of Cu^2+^ doping, the double-doped CeO_2_ nanospheres obviously underwent a morphology transformation from hollow to core–shell, and then to be solid. The high-resolution TEM image in Fig. 3 shows that each Cu_*x*_Co_1–*x*_–CeO_2_–Pt nanosphere is decorated by hundreds of ultra-small Pt nanoparticles (less than 2 nm) and the Cu_*x*_Co_1–*x*_–CeO_2_ nanospheres are composed of closely packed CeO_2_ nanoparticles with diameters of about 5 nm as primary building blocks. The clearly observed lattice spacing listed in Table S3† agrees well with that of Pt (200) (0.196 nm) and CeO_2_ (111) (0.312 nm). The high angle annular dark field scanning transmission electron microscopy (HAADF-STEM) images of Cu_0.66_Co_0.34_–CeO_2_–Pt demonstrate the evenly distributed Cu, Pt and Co elements, as shown in Fig. 4.

**Fig. 5:**
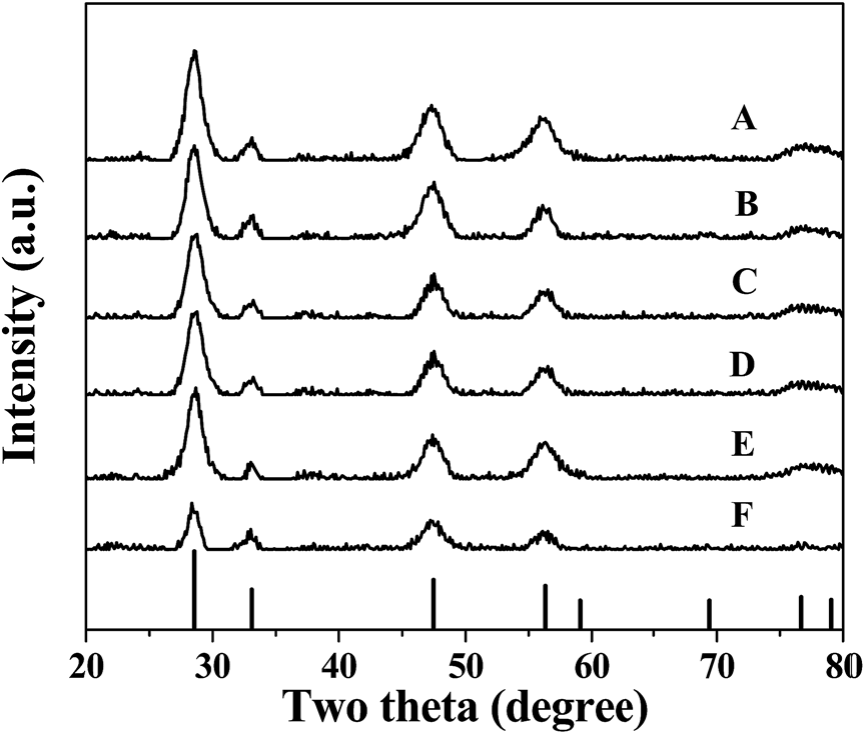
XRD patterns of (A) Cu–CeO_2_–Pt; (B) Cu_0.66_Co_0.34_–CeO_2_–Pt; (C) Cu_0.50_Co_0.50_–CeO_2_–Pt; (D) Cu_0.34_Co_0.66_–CeO_2_–Pt; (E) Co–CeO_2_–Pt; (F) CeO_2_–Pt.

In the X-ray diffraction (XRD) patterns of the Cu_*x*_Co_1–*x*_–CeO_2_–Pt and CeO_2_–Pt nanospheres (Fig. 5), the diffraction peaks of all the products can be indexed to a pure phase of fcc CeO_2_ structures (JCPDS no. 34-0394). The peaks at 2*θ* = 28.549°, 33.077°, 47.483°, 56.342°, 59.09°, 69.416°, 76.704° and 79.077° correspond to the characteristic (111), (200), (220), (311), (222), (400), (331) and (420) reflections, respectively. No signals related to impurities, such as cobalt oxide or copper oxide, can be found for all samples, indicating the homogeneous doping of Cu and Co ions in the CeO_2_ solid solutions. The contents of the elements Cu, Co, Ce and Pt for the Cu_*x*_Co_1–*x*_–CeO_2_–Pt nanospheres were then determined by ICP-MS analysis and are listed in Table S4.† It can be seen that the loading amounts of Pt (mol%) are less than 1.4% if Cu^2+^ is introduced, however, in the absence of Cu^2+^, this percentage increased to 4.1% for Co–CeO_2_–Pt and 4.8% for CeO_2_–Pt, which were much higher than the others. On the contrary, Co^2+^ doping has little effect on the deposition amount of Pt.

**Fig. 6:**
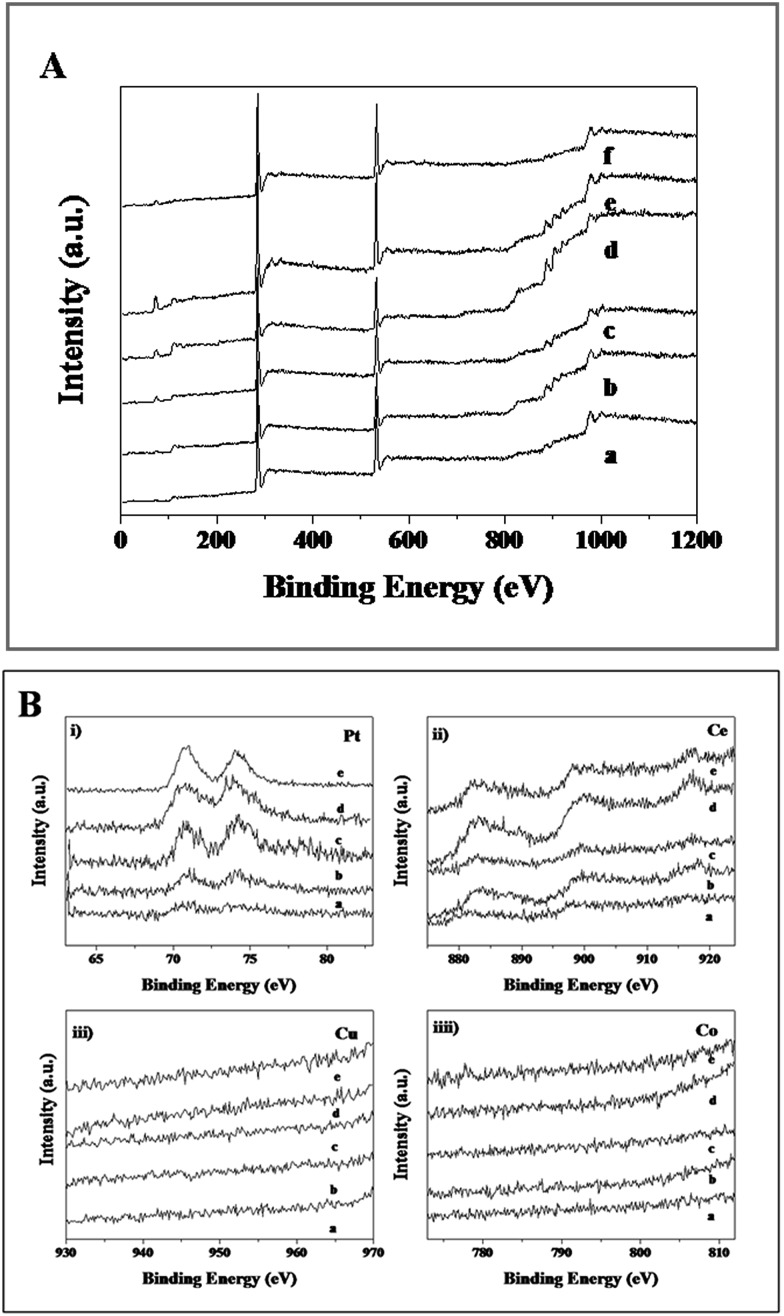
(A) XPS spectra of (a) Cu–CeO_2_–Pt, (b) Cu_0.66_Co_0.34_–CeO_2_–Pt, (c) Cu_0.50_Co_0.50_–CeO_2_–Pt, (d) Cu_0.34_Co_0.66_–CeO_2_–Pt, (e) Co–CeO_2_–Pt, (f) CeO_2_–Pt. (B) XPS spectra of (a) Cu–CeO_2_–Pt; (b) Cu_0.66_Co_0.34_–CeO_2_–Pt; (c) Cu_0.50_Co_0.50_–CeO_2_–Pt; (d) Cu_0.34_Co_0.66_–CeO_2_–Pt; (e) Co–CeO_2_–Pt.

XPS analysis was employed to determine the surface elements and their valence states of the Cu_*x*_Co_1–*x*_–CeO_2_–Pt and CeO_2_–Pt nanospheres (Fig. 6). All of the samples show characteristic peaks at the binding energies of 71.3 eV (Pt 4f_7/2_) and 74.7 eV (Pt 4f_5/2_) of the element Pt, and the two peaks at 882.8 and 899.5 eV correspond well to the Ce 3d_5/2_ and Ce 3d_3/2_ spin–orbit peaks of CeO_2_. As previously reported, the XPS spectrum of Co 2p shows two major peaks at 795.5 and 780.4 eV, corresponding to Co 2p_1/2_ and Co 2p_3/2_ spin–orbit coupling, respectively.^46^ Two major peaks lying at 932.2 and 954.2 eV are characteristic signals of Cu^2+^ with Cu 2p_3/2_ and Cu 2p_1/2_ orbits, respectively.^46^ Unfortunately, due to the low doping content of Co and Cu in Cu_*x*_Co_1–*x*_–CeO_2_–Pt, no Co and Cu signals can be detected in all the samples. However, in combination with the HAADF-STEM characterization and ICP results, the successful formation of Cu_*x*_Co_1–*x*_–CeO_2_–Pt hybrids can be confirmed.

**Fig. 7:**
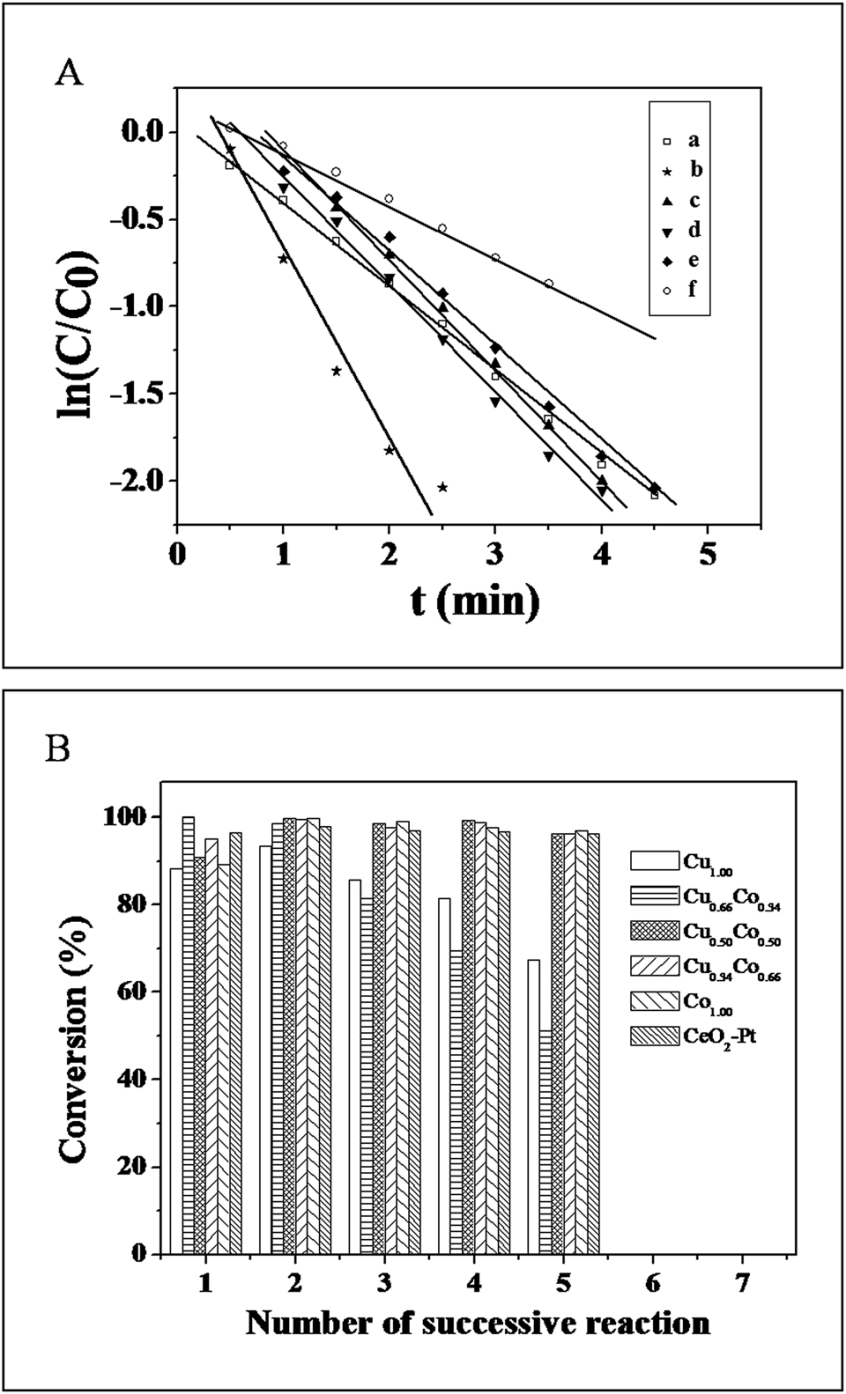
(A) ln(*C*/*C*_0_) *versus t* for the reduction of 4-NP catalyzed by (a) Cu–CeO_2_–Pt; (b) Cu_0.66_Co_0.34_–CeO_2_–Pt; (c) Cu_0.50_Co_0.50_–CeO_2_–Pt; (d) Cu_0.34_Co_0.66_–CeO_2_–Pt; (e) Co–CeO_2_–Pt; (f) CeO_2_–Pt. (B) Conversion in successive reaction cycles of Cu_*x*_Co_1–*x*_–CeO_2_–Pt and CeO_2_–Pt.

In the following, the reduction of 4-NP to 4-AP by NH_3_BH_3_ was selected to evaluate the catalytic performance of the as-obtained Cu_*x*_Co_1–*x*_–CeO_2_–Pt and CeO_2_–Pt samples. As is known, 4-NP exhibits a strong characteristic absorption peak at 317 nm while at pH < 7, but it will be ionized as the alkalinity of the solution increases, resulting in a spectral shift to 400 nm.^35^ In our case, the absorption peak of 4-NP remained at 317 nm despite adding NH_3_BH_3_ solution, indicating that the NH_3_BH_3_ molecules were stable enough in water in the absence of the catalysts.^34^ However, after addition of the Cu_*x*_Co_1–*x*_–CeO_2_–Pt hybrids, the absorption intensity at 400 nm gradually increased. The color of the reaction system changed from bright yellow to colorless. Since NH_3_BH_3_ was in a large excess relative to 4-NP, its concentration could be considered as constant during the reaction period. Thus, the reduction rate can be evaluated by pseudo-first-order kinetics with respect to 4-NP. Fig. 7A shows ln(*C*/*C*_0_) *versus* reaction time *t*, which was obtained from the relative intensity ratio of the absorbance (*A*/*A*_0_) at 400 nm. Here, *C*_0_ and *C* represent the initial and instantaneous concentrations of 4-NP, respectively; and *k* and *t* stand for the rate constant and the reaction time in turn. As all of these plots followed first-order reaction kinetics very well, the value *k* can be calculated from the equation ln(*C*/*C*_0_) = *kt* (Table 1). The catalytic activity of the six samples follows this sequence: Cu_0.66_Co_0.34_–CeO_2_–Pt > Cu_0.50_Co_0.50_–CeO_2_–Pt > Cu–CeO_2_–Pt > Cu_0.34_Co_0.66_–CeO_2_–Pt > Co–CeO_2_–Pt > CeO_2_–Pt, showing a strong dependence on the composition of Cu and Co. Furthermore, the turnover frequency (TOF), defined as moles of the reactant 4-NP converted per mole of active metal in the catalyst per hour was also calculated (Table 1). Though the most stable sample of Cu_0.50_Co_0.50_–CeO_2_–Pt shows a moderate catalytic activity compared to others for catalytic 4-NP conversion, its TOF of 480 h^–1^ is still at least five times higher than our previously reported Pt@CeO_2_ catalysts.^34^

**Table 1:** Comparison of the rate constant *k*, TOF and conversion for 4-NP reduction with Cu_*x*_Co_1–*x*_–CeO_2_–Pt and CeO_2_–Pt catalysts^*a*^

Sample	*k* (s^–1^)	*M*_Pt_ (mmol)	*M*_4-NP_ (mmol)	*t* (min)	TOF (h^–1^)	*P* (%)	Ref
Cu_1.00_–CeO_2_–Pt	0.451	3.35 × 10^–6^	2.00 × 10^–4^	3.83	935	80	This work
Cu_0.66_Co_0.34_–CeO_2_–Pt	0.995	7.24 × 10^–6^	2.00 × 10^–4^	1.88	882	51.2	This work
Cu_0.50_Co_0.50_–CeO_2_–Pt	0.633	7.22 × 10^–6^	2.00 × 10^–4^	3.46	480	98.8	This work
Cu_0.34_Co_0.66_–CeO_2_–Pt	0.614	8.24 × 10^–6^	2.00 × 10^–4^	3.17	459	99.2	This work
Co_1.00_–CeO_2_–Pt	0.555	2.05 × 10^–5^	2.00 × 10^–4^	3.66	160	97.8	This work
CeO_2_–Pt	0.285	2.09 × 10^–5^	2.00 × 10^–4^	6.15	93	89.9	This work
Pt@CeO_2_	—	1.00 × 10^–4^	1.00 × 10^–3^	—	46	—	34
Pt@CeO_2_/RGO	—	1.00 × 10^–4^	1.00 × 10^–3^	—	90	—	34

^*a*^ *M*_Pt_: mole of noble metals; *M*_4-NP_: mole of 4-NP; *t*: conversion time; *P*: conversion percentage.

Good reproducibility and stability are also important for the evaluation of catalysts. In order to avoid the loss of the Cu_*x*_Co_1–*x*_–CeO_2_–Pt catalysts caused by the separation process, cycling tests have been conducted *in situ*. Fig. 7B shows the conversion in successive reaction cycles of the Cu_*x*_Co_1–*x*_–CeO_2_–Pt catalysts, and the conversions of 4-NP for the fifth cycle are listed in Table 1. It is found that the stability of the samples varied with the compositions of different doping ions, and that the Co rich samples show better stability among these catalysts.

**Fig. 8:**
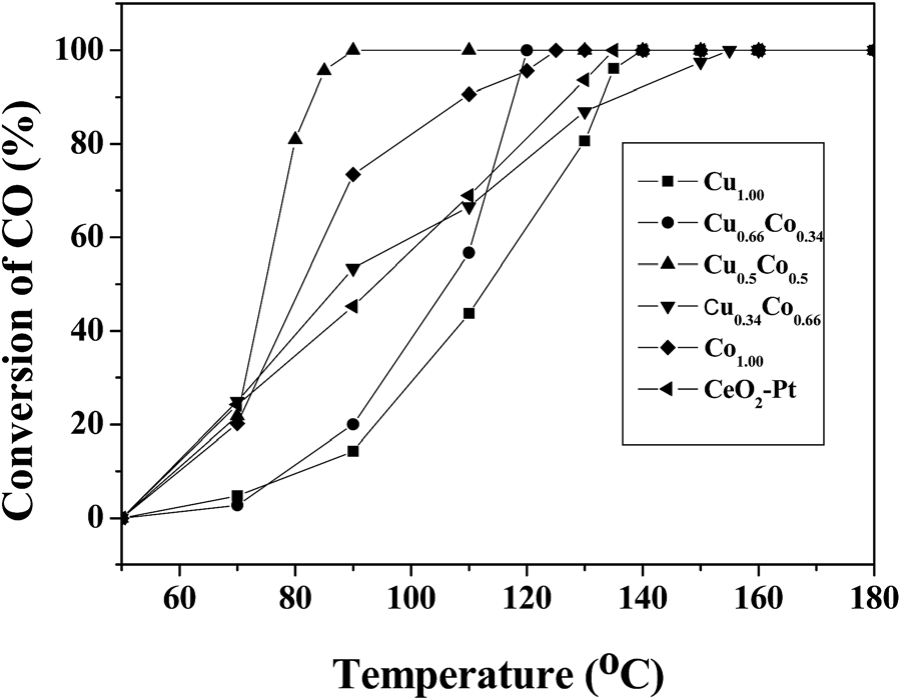
CO conversion curves of Cu_*x*_Co_1–*x*_–CeO_2_–Pt and CeO_2_–Pt.

Besides, catalytic CO oxidation was employed to evaluate these catalysts. In the catalytic process, the gas mixture of CO and O_2_ was introduced into the inner space of a stainless steel reaction tube filled with Cu_*x*_Co_1–*x*_–CeO_2_–Pt catalysts. *T*_100_, the temperature for 100% CO oxidation, is used to compare the catalytic activity of these samples. Despite of the minor difference in the doped components, these samples show quite different catalytic performance. Fig. 8 presents their CO conversion curves, and the *T*_100_ values follow a sequence of: Cu_0.50_Co_0.50_–CeO_2_–Pt (90 °C) < Cu_0.66_Co_0.34_–CeO_2_–Pt (120 °C) < Co–CeO_2_–Pt (125 °C) < CeO_2_–Pt (135 °C) < Cu–CeO_2_–Pt (140 °C) < Cu_0.34_Co_0.66_–CeO_2_–Pt (155 °C). It can be seen that the catalytic performance of our samples improves with the increase in the doping amount of Co until the ratio of Cu^2+^/Co^2+^ = 0.5/0.5 is reached, and then this deteriorates quickly upon the doping of more Co^2+^ ions. This result indicates that Cu^2+^ and Co^2+^ ions can replace the tetravalent Ce^4+^ in the CeO_2_ fluorite lattice to produce more oxygen vacancies, but an appropriate doping ratio of Cu^2+^/Co^2+^ ions is needed in order to realize optimal catalytic performance in our case.

## Conclusions

We have successfully prepared a series of Cu_*x*_Co_1–*x*_–CeO_2_–Pt hybrid nanospheres. Based on detailed characterization including SEM, TEM, XPS, ICP and XRD, it is found that (I) in the nucleation process, the introduction of Cu^2+^ accelerated the nucleation rate compared with CeO_2_, leading to the formation of the smaller sized CeO_2_. (II) The Cu^2+^ doping concentration can affect the amount of Pt nanoparticles deposited to a greater extent than Co^2+^. With the increase in Cu^2+^ doping concentration, the amount of Pt nanoparticles on the Cu_*x*_Co_1–*x*_–CeO_2_ nanospheres decreases. However, the related effect of Cu^2+^ ions was quite small. (III) The high purity of all products indicates the formation of homogeneous Ce–Cu–Co–O, Ce–Cu–O or Ce–Co–O solid solutions.

Based on the catalytic tests, it is also found that the doping amount of Cu^2+^ greatly influences the catalytic performance of the Cu_*x*_Co_1–*x*_–CeO_2_ nanospheres. Though the most stable sample of Cu_0.50_Co_0.50_–CeO_2_–Pt shows a moderate catalytic activity compared to others for catalytic 4-NP conversion, its TOF of 480 h^–1^ is still much higher than our previously reported Pt@CeO_2_ catalysts. Moreover, among the Cu_*x*_Co_1–*x*_–CeO_2_–Pt samples, the Cu_0.50_Co_0.50_–CeO_2_–Pt nanospheres exhibited the best catalytic activity, attaining 100% CO conversion at 90 °C, which is also higher than previously reported Pt catalysts. It can be anticipated that this kind of double doped nanocatalysts will have great potential for application.

## Supplementary Material

Supplementary information
