# Supplementary material for: Decoration of Pt on Cu/Co double-doped CeO2 nanospheres and their greatly enhanced catalytic activity
Source: Chem Sci. 2015 Nov 25;7(3):1867–73. doi: 10.1039/c5sc04069h (PMC5965059; doi:10.1039/c5sc04069h)
Supplement: Supplementary file 1 [file SC-007-C5SC04069H-s001.pdf]

Electronic Supplementary Material (ESI) for Chemical Science.
This journal is © The Royal Society of Chemistry 2015

Supporting Information

Decoration of Pt on Cu/Co Double-Doped CeO_2 Nanospheres and Their Greatly Enhanced Catalytic Activity

Fan Wang,[b] Wang Li,[a] Xilan Feng,[a] Dapeng Liu,*[a] and Yu Zhang*[a]

a) Key Laboratory of Bio-Inspired Smart Interfacial Science and Technology of Ministry of Education, School of Chemistry and Environment, Beihang University, Beijing 100191, P.R. China

b) State Key Laboratory of Rare Earth Resource Utilization, Changchun Institute of Applied Chemistry, Chinese Academy of Sciences, Changchun, 130022 Jilin, China.

Sample	CeO_2	Cu-CeO_2	$Cu_{0.66}Co_{0.34}$-CeO_2	$Cu_{0.50}Co_{0.50}$-CeO_2	$Cu_{0.34}Co_{0.66}$-CeO_2	Co-CeO_2
**Average Size (nm)**	174.03	68.53	100.37	207.9	224.37	194.9

Table S1. Average size of Cu_xCo_{1-x}-CeO_2 and pure CeO_2 nanospheres.

Sample	CeO_2-Pt	Cu-CeO_2-Pt	$Cu_{0.66}Co_{0.34}$-CeO_2-Pt	$Cu_{0.50}Co_{0.50}$-CeO_2-Pt	$Cu_{0.34}Co_{0.66}$-CeO_2-Pt	Co-CeO_2-Pt
**Average Size (nm)**	181.22	65.02	114.31	181.59	205.33	177.29

Table S2. Average size of Cu_xCo_{1-x}-CeO_2-Pt and CeO_2-Pt nanospheres.

**Sample**	**CeO_2-Pt**	**Cu-CeO_2-Pt**	**$Cu_{0.66}Co_{0.34}$-CeO_2-Pt**	**$Cu_{0.50}Co_{0.50}$-CeO_2-Pt**	**$Cu_{0.34}Co_{0.66}$-CeO_2-Pt**	**Co-CeO_2-Pt**
**Lattice spacing CeO_2 (111)**	0.300 nm	0.324 nm	0.317 nm	0.303 nm	0.321 nm	0.316 nm
**Lattice spacing Pt (200)**	0.192 nm	0.196 nm	0.194 nm	0.195 nm	0.194 nm	0.195 nm

Table S3. Lattice spacing of Cu_xCo_{1-x}-CeO_2-Pt and CeO_2-Pt nanospheres.

Sample	Co (mg/kg)	Cu (mg/kg)	Pt (mg/kg)	Ce (mg/kg)	Pt (mol%)	Cu (mol%)	Co (mol%)	Ce (mol%)	Cu/Co in molar ratio
**Cu-CeO_2-Pt**	—	3066	6535	467600	1.0	1.4	—	97.6	—
**$Cu_{0.66}Co_{0.34}$-CeO_2-Pt**	1895	4777	14120	773800	1.3	1.3	0.6	96.8	2.3
**$Cu_{0.50}Co_{0.50}$-CeO_2-Pt**	2883	4357	14080	767000	1.3	1.2	0.9	96.6	1.4
**$Cu_{0.34}Co_{0.66}$-CeO_2-Pt**	3628	2550	16080	822800	1.4	0.7	1.0	97.0	0.6
**Co-CeO_2-Pt**	7217	—	39950	647300	4.1	—	2.5	93.4	—
**CeO_2-Pt**	—	—	40770	577600	4.8	—	—	95.2	—

Table S4. ICP-MS analyses of CeO_2-Pt and Cu_xCo_{1-x}-CeO_2-Pt nanospheres.
